# COVID-19 mortality and ICU admission: the Italian experience

**DOI:** 10.1186/s13054-020-02957-9

**Published:** 2020-05-15

**Authors:** Paolo Immovilli, Nicola Morelli, Elio Antonucci, Guido Radaelli, Mario Barbera, Donata Guidetti

**Affiliations:** 1 Neurology Unit, Emergency Department, Guglielmo da Saliceto Hospital, via Taverna 39, 29121 Piacenza, Italy; 2 Radiology Unit, Radiology Department, Guglielmo da Saliceto Hospital, Piacenza, Italy; 3 Intermediate Care Unit, Emergency Department, Guglielmo da Saliceto Hospital, Piacenza, Italy

**Keywords:** COVID-19, ICU, SARS-CoV-2

To the Editor,

We read with great interest the article by Li et al. highlighting ten issues regarding COVID-19 critical care management, the first of which regarding intensive care unit (ICU) capability to face SARS-CoV-2 pandemic [1].

On February 21st, the first COVID-19 patient was identified in the province of Lodi; since then, Italy is facing one of the biggest outbreaks in the world, accounting for more than 100,000 cases, 11,000 infected patients hospitalized and 1300 patients sent to the ICU, leading to more than 12,000 deaths as of March 31st [2].

Lombardy had 1006 patients requiring advanced respiratory support on March 19th and a standard operational capacity of 724 ICU beds [3].

The magnitude of the outbreak was exceptional in Lombardy, Emilia-Romagna, and Veneto, and it was milder in some other regions, so we compared data of Protezione Civile on March 31st on mortality and ICU admission between Italian regions, accounting for different ICU capabilities with respect to different outbreak magnitudes [3].

Data from 20 regions were collected; the average interregional case fatality rate (CFR) was 7.5%, range 3.1–16.7%, and the average ICU admission rate was 21.4%, range 9.4–45.9%. A statistically significant negative correlation was observed between the CFR and ICU admission rate (Pearson’s *r* − 0.53, *p* value 0.014) and *R*^2^ was 0.24, suggesting an association between mortality and the absence of treatment in ICU (Fig. 1).

**Fig. 1:**
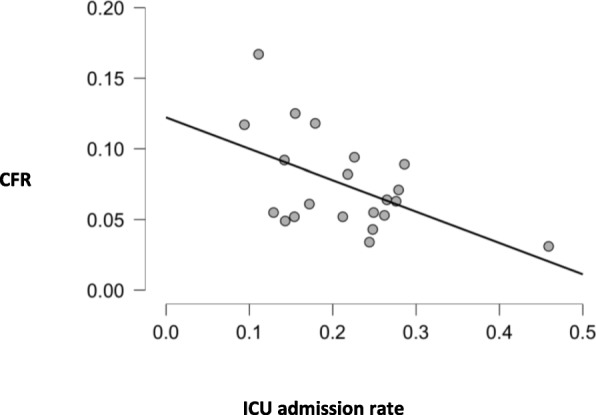
Case fatality rate (CFR) and intensive care unit (ICU) admission rate are plotted for 20 Italian regions according to the data of Protezione Civile on March 31, 2020

The analysis of mortality during an outbreak is no easy feat and a precise evaluation can be obtained only once the outbreak is over. Furthermore, the high Italian mortality may well be attributable to a large proportion of elderly persons in the Italian population, to an ascertainment bias and/or diagnosis bias, leading to an underestimation of the milder cases and mortality overestimation.

However, examining the differing outbreak magnitudes in regions with different ICU availability evidenced a discrepancy in the percentage of ICU-admitted patients. Indeed, there was a higher mortality rate in the northern region where fewer patients could be admitted into an ICU. These preliminary data evidence the pivotal preventive role played by early lockdown measures to reduce outbreak magnitude and place less pressure on ICU beds availability; however, these data should be interpreted with caution because of possible bias: patients could be allowed outside the ICU due to various reasons (i.e., age, comorbidities, frailty index), as it occurs in daily clinical practice.

## Data Availability

Data published online by Italian Civil Protection Department (http://opendatadpc.maps.arcgis.com/apps/opsdashboard/index.html#/b0c68bce2cce478eaac82fe38d4138b1; seen on March 31, 2020).
